# A novel fusion tool to enable G protein-coupled receptor structure determination

**DOI:** 10.1107/S2059798326003785

**Published:** 2026-05-15

**Authors:** Nita R. Shah, Mathieu Oosterlaken, Claudine Bisson, Mattia Bertinelli, Alicia M. Churchill-Angus, Andrew Hutchin, Jola Kopec, Vadim Kotov, Ciaran R. McFarlane, Erika Griss Pascualli, Ana Pavic, Matthias Zebisch, Cédric Fiez-Vandal, Edoardo Fabini, Stéphanie Duclos

**Affiliations:** a Evotec (United Kingdom) Innovation Drive, Milton Park AbingdonOX14 4RZ United Kingdom; b Evotec (Germany) Essener Bogen 7 22419Hamburg Germany; National Centre for Biological Sciences-TIFR, India

**Keywords:** G protein-coupled receptors, cryo-EM, fusion tags, fiducial tags

## Abstract

Structure determination of G protein-coupled receptors (GPCRs) plays an important role in accelerating drug development against this medically important protein family. This study outlines the development of a new fusion tool to enable structure determination of GPCRs in inactive conformations by cryo-EM. Initially, a PDB mining approach was applied to select eight naturally occurring proteins with the intention of fusing them into the intracellular loop 3 (ICL3) of GPCRs to create a suitable fiducial marker for cryo-EM workflows. During the selection process, candidates with known high-affinity protein binders were prioritized to enable a further increase in the protein mass of the fiducial marker. Fusion constructs were generated with adenosine receptor A_2A_ (A_2A_R) and were assessed for expression and aggregation levels. For the two best-performing new fusion constructs, ligand binding was characterized to ensure that the fusion tag did not significantly affect protein behaviour. A_2A_R with a β-lactamase fusion in ICL3 and binding partner β-lactamase inhibitory protein II (BLIPII) was then selected to solve an antagonist-bound structure. The overall map was resolved to an average of 3.2 Å resolution with continuous helices connecting the β-lactamase to helices 5 and 6 of A_2A_R. Focused refinement of the A_2A_R region improved the local resolution and map detail in the orthosteric site, thereby allowing confident modelling of the antagonist ligand, ZM241385, which matches previously described X-ray crystallographic structures. This new fusion provides an alternative option for GPCR structure determination, with several potential benefits compared with existing tools, such as a more favourable position relative to the GPCR to reduce potential clashes.

## Abstract 2

The development of a new fusion tool in which a β-lactamase fusion tag and a binding partner enabled the structure determination of an antagonist-bound G protein-coupled receptor by cryo-EM is reported.

## 1. Introduction

G protein-coupled receptors (GPCRs) are targeted by about one third of the small-molecule drugs currently on the market (Santos *et al.*, 2017 ▸). Structural insights can streamline drug development via structure-based drug-design workflows. A crucial component of this process is determining experimental structures of ligand-bound GPCRs to identify binding poses and support further chemical design throughout the design–make–test–analyze (DMTA) cycle. However, GPCRs have historically been challenging targets for structure solutions, especially considering their small size, flexible nature and membrane-embedded native state.

GPCRs share a conserved architecture consisting of seven transmembrane (TM) helices, an eighth helix running parallel to the lipid bilayer and multiple intracellular and extracellular loops that connect the TM segments (Palczewski *et al.*, 2000 ▸; Rasmussen *et al.*, 2007 ▸). Historically, GPCR structures were determined by X-ray crystallography, often requiring fusion tags, binding partners or stabilizing mutations to facilitate crystallization (Cherezov *et al.*, 2007 ▸; Warne *et al.*, 2008 ▸; Hollenstein *et al.*, 2013 ▸; Jazayeri *et al.*, 2016 ▸, 2017 ▸; Oswald *et al.*, 2016 ▸; Cheng *et al.*, 2017 ▸; Robertson *et al.*, 2018 ▸; Chun *et al.*, 2012 ▸). More recently, cryo-EM has become the dominant method for GPCR structure determination, partly due to requiring a lower yield and no longer depending on crystal formation (García-Nafría & Tate, 2021 ▸). Agonist-bound GPCR complexes, in which a ligand-bound GPCR engages G protein subunits, are well suited for cryo-EM because the large, rigid G protein complex stabilizes the receptor and facilitates particle alignment. The development of several methods that stabilize the G protein complex has supported agonist-bound GPCR structure solutions (Errey & Fiez-Vandal, 2020 ▸; Duan *et al.*, 2020 ▸). In contrast, antagonist-bound GPCRs lack G protein binding partners and therefore present a major challenge: their small size (∼40–50 kDa) and flexible loops do not provide sufficient mass or rigidity for cryo-EM data processing. Strategies have been developed to increase the protein mass and introduce fiducial markers (Singh *et al.*, 2026 ▸). One widely used approach is the insertion of fusion tags into intracellular loop 2 (ICL2) or ICL3 (Tsutsumi *et al.*, 2020 ▸). For example, fusion of a thermostabilized apocyto­chrome b562 (bRIL) tag into ICL3 with continuous helices connecting the GPCR and bRIL can enforce rigidity. The bRIL tag can be enlarged with protein binders: a Fab that binds bRIL and a VHH that binds and rigidifies the Fab hinge region, together adding ∼75 kDa of mass and enabling high-resolution cryo-EM reconstructions (Mukherjee *et al.*, 2020 ▸; Tsutsumi *et al.*, 2020 ▸; Ereño-Orbea *et al.*, 2018 ▸; Gao *et al.*, 2025 ▸). However, the bRIL fusion strategy is not universally applicable; variations in TM-helix length and intracellular loop sizes may hinder rigid integration of bRIL while maintaining receptor folding. This limitation was highlighted by Guo and coworkers, who demonstrated that truncating the loop between helices 2 and 3 of bRIL improved the stability of a fusion with β_2_-adrenoceptor (β_2_AR), likely by reducing steric clashes at the cytosolic face of the receptor (Guo *et al.*, 2024 ▸).

In this study, we describe a new fusion tag that can be used to solve the structure of antagonist-bound GPCRs by cryo-EM. We initially assessed protein quality and yield for an array of fusion-tag candidates and subsequently confirmed that the addition of various tags did not alter ligand binding. Having selected our lead fusion candidate, we demonstrated that the tag, in complex with its binding partner, can be used to solve a high-resolution structure of a GPCR and resolve a small-molecule antagonist. This new tag expands the available range of tools for GPCR structural biology and thereby supports drug discovery for this prominent class of receptors.

## 2. Materials and methods

### 2.1. Fusion-tag selection and construct design

Fusion-tag candidates were initially identified through both manual and computational mining of proteins or protein domains from the Protein Data Bank (PDB). For both search methods, proteins were considered based on size and the presence of N- and C-terminal helices with desired orientations relative to one another (*i.e.* close to antiparallel with a separation of ∼10 Å). Computational mining of PDB entries using the desired selection criteria involved removing ligand and water molecules, retrieving α-helices based on secondary-structure annotation, selecting structures containing individual chains with N- and C-terminal helices (minimum helix length of eight residues, with a maximum of five residues separation from the termini), measuring the distance between the C^α^ atoms of the N- and C-termini of the terminal helices and measuring the angle between the N- and C-terminal helices. PDB manipulations were completed with *GEMMI* (Wojdyr, 2022 ▸). Fusion-tag candidates were then evaluated based on protein and structure characteristics. Membrane proteins, homo-oligomeric proteins and artificially designed proteins were removed from the list. Proteins were prioritized if they originated from thermophilic organisms and if there were structures in the PDB of the candidate tag in complex with a protein binding partner. Literature documentation of protein expression and purification was used to assess protein solubility and stability.

The shortlist of fusion-tag candidates was assessed based on their propensity to form continuous helical connections when inserted into the ICL3 of adenosine A_2A_ receptor (A_2A_R) by *in silico* methods. Tag sequences were inserted into the ICL3 with different entry and exit points and predicted structural models of each fusion construct were generated by *AlphaFold*2 (Jumper *et al.*, 2021 ▸). These predicted models were analyzed for continuous helical connections, confidence scores and alignment scores with the inactive conformation of A_2A_R (for example PDB entry 5iu4; Segala *et al.*, 2016 ▸). The connection regions of some fusion proteins as well as the fusion tags themselves were further optimized with rationally designed mutations using predicted structural models and were assessed *in silico* in a similar manner (Supplementary Tables S1 and S3).

All A_2A_R expression vectors contain the same expression cassette, with an N-terminal influenza hemagglutinin (HA) signal sequence and FLAG tag, C-terminal tobacco etch virus (TEV) protease cleavage site, green fluorescent protein (eGFP A206K) and 10×His-tag, and A_2A_R from residues 2 to 316. The control construct has a bRIL tag inserted in place of residues 235–244 of A_2A_R, connecting to TM5 and TM6. The final list of A_2A_R constructs with different fusion tags inserted into ICL3 are listed in Supplementary Table S1. All constructs were codon-optimized for insect-cell expression and cloned into pFastBac-1 or pFastBac Dual (Fusion 8). For Fusion 8, RagA was inserted into the ICL3 of A_2A_R and co-expressed with a binding partner, the GTPase domain of RagC (residues 59–376, with an N-terminal 6×His-tag, as described in Supplementary Table S1). Residues 40–311 of BLIPII from *Streptomyces exfoliatus* (UniProt ID O87916) were cloned into pET-29b with an N-terminal 10×His-tag.

### 2.2. Expression

For small-scale experiments, A_2A_R fusion constructs were expressed in *Spodoptera frugiperda* Sf21 cells in 40 ml Sf900-II medium at 27°C for 72 h post-infection. For large-scale expression of A_2A_R fusion constructs, 10 l of cells were harvested 65 h post-infection.

BLIPII was expressed in *Escherichia coli* BL21 (DE3) cells in 5 l LB (Teknova) medium induced at 18°C with 1 m*M* isopropyl β-d-1-thiogalactopyranoside for 18 h.

### 2.3. Purification

For small-scale experiments, the harvested cells were solubilized in 20 m*M* HEPES pH 7.5, 100 m*M* NaCl, 1%(*w*/*v*) laurylmaltose neopentylglycol (LMNG), 0.1%(*w*/*v*) cholesteryl hemisuccinate (CHS) and protease inhibitors for 1–2 h at 4°C. Insoluble material was pelleted and the solubilized material was pulled down by incubation with FLAG affinity resin at 4°C for 2 h. The affinity resin was then washed with 20 m*M* HEPES pH 7.5, 100 m*M* NaCl, 0.1%(*w*/*v*) LMNG, 0.01%(*w*/*v*) CHS and eluted with the same buffer supplemented with 0.25 mg ml^−1^ FLAG peptide. The eluates were analyzed by SDS–PAGE, Western blot with anti-FLAG primary antibody and fluorescence size-exclusion chromatography (fSEC) in 20 m*M* HEPES pH 7.5, 100 m*M* NaCl, 0.1%(*w*/*v*) LMNG, 0.01%(*w*/*v*) CHS. The data were adjusted to account for the difference in path length between the UV detector [UV signal for the standards (Std)] and the fluorescence detector (fSEC signal for the A_2A_R fusion proteins).

Large-scale purifications of A_2A_R fusion supplied protein for SPR and cryo-EM experiments. The cell pellets were lyzed with a Dounce homogenizer in 40 m*M* HEPES pH 7.5, 250 m*M* NaCl, 1 m*M* TCEP, benzonase (10 U ml^−1^) and protease inhibitors [cOmplete EDTA-free, Protease Inhibitor Cocktail (Roche)] followed by solubilization using LMNG and CHS to final concentrations of 0.5%(*w*/*v*) and 0.05%(*w*/*v*), respectively, with gentle agitation for 2 h at 4°C. Clarified supernatant was incubated with anti-FLAG M2 affinity resin (Sigma) for 2 h at 4°C followed by washing with 40 m*M* HEPES pH 7.5, 250 m*M* NaCl, 0.1 m*M* TCEP, 0.01%(*w*/*v*) LMNG, 0.001%(*w*/*v*) CHS and protein was eluted into this buffer supplemented with 0.4 mg ml^−1^ FLAG peptide. For SPR experiments, the eluted protein was further purified by size-exclusion chromatography (SEC) on a Superdex 200 10/300 column with 40 m*M* HEPES pH 7.5, 250 m*M* NaCl, 0.1 m*M* TCEP, 0.01%(*w*/*v*) LMNG, 0.001%(*w*/*v*) CHS. For cryo-EM experiments, A_2A_R fusion protein eluted from the affinity resin was complexed with BLIPII (1:1.2 molar ratio) and 10 µ*M* ZM241385 for 1 h at 4°C. The complex was then further purified by SEC on a Superose 6 3.2/300 column with 40 m*M* HEPES pH 7.5, 250 m*M* NaCl, 0.01%(*w*/*v*) LMNG, 0.001%(*w*/*v*) CHS, 1 µ*M* ZM241385.

Cells expressing BLIPII were lyzed by sonication in 40 m*M* HEPES pH 7.5, 250 m*M* NaCl, 5%(*v*/*v*) glycerol, protease inhibitors and benzonase (10 U ml^−1^). Clarified lysate was applied onto a HisTrap Excel column. The column was washed with 40 m*M* HEPES pH 7.5, 250 m*M* NaCl, 5%(*v*/*v*) glycerol, 1 m*M* TCEP followed by elution with a gradient of this buffer with 0–500 m*M* imidazole. Eluted BLIPII was then further purified by SEC on a Superdex 200 26/600 column with running buffer consisting of 40 m*M* HEPES pH 7.5, 250 m*M* NaCl, 5%(*v*/*v*) glycerol, 1 m*M* TCEP.

All purified proteins were characterized by SDS–PAGE analysis, Western blot and fSEC. BLIPII was further characterized by intact mass spectrometry.

### 2.4. Surface plasmon resonance (SPR)

SPR binding experiments were performed on a Biacore T200 optical biosensor unit (Cytiva Life Sciences) at 10°C in 50 m*M* HEPES pH 7.5, 150 m*M* NaCl, 50 µ*M* EDTA, 0.01%(*w*/*v*) LMNG, 0.001%(*w*/*v*) CHS, 3% DMSO; DMSO was omitted during the immobilization steps. The sample compartment was set at 10°C during the immobilization procedure to preserve sample integrity. All tool compounds used in the study were purchased from Tocris Bioscience.

A_2A_R proteins were immobilized on a NiHC1500M sensor chip (XanTec bioanalytics GmbH). All of the immobilization procedure was conducted at a 10 µl min^−1^ flow rate. The surface was conditioned using 350 m*M* EDTA pH 8.0 for 420 s, followed by a 300 s injection of 500 µ*M* NiCl_2_. The sensor chip was then activated using a 1:1 mixture of 400 m*M* 1-ethyl-3-(3-dimethylaminopropyl)carbodiimide (EDC) and 100 m*MN*-hydroxysuccinimide (NHS) for 900 s, immediately followed by an injection of protein at 50 µg min^−1^ for 1200 s, resulting in a density of 5000–7000 response units (RU) for each protein. Finally, the surface was deactivated with 1 *M* ethanolamine–HCl pH 8.5 injected in the reference channel only for 420 s.

Interaction analyses were performed using a 50 µl min^−1^ flow rate. For ANR94, KW3902, PSB0777 and LUF5834 analysis was performed using the multi-cycle kinetics mode with a 60 s association time and a 200 s dissociation time, injecting each sample at 0.004, 0.008, 0.016, 0.031, 0.063, 0.125, 0.25, 0.5, 1 and 2 µ*M*. For ZM241385, single-cycle kinetics mode was performed using a 120 s association time and 1800 s dissociation time at the following serial dilutions: 0.012, 0.037, 0.111, 0.333 and 1 µ*M*. The affinity and kinetics were obtained using the *BIAevaluation* software, fitting the data to a 1:1 interaction model.

### 2.5. Cryo-EM

The size-exclusion chromatography (SEC) peak fraction of A_2A_R-β-lactamase_Bli_ complexed with BLIPII and the antagonist ligand ZM241385 was frozen on UltrAuFoil mesh 300 1.2/1.3 grids at 0.38 mg ml^−1^ using a Vitrobot Mark IV with standard blotting conditions (3 s blot time, −8 blot force). Grids were assessed for ice and particle quality on a Titan Krios microscope (Thermo Fisher Scientific) with a K3 camera (Gatan), followed by the collection of 10 931 micrograph movies at 105 000 nominal magnification (0.835 Å per pixel). The data were collected with a defocus range from −1.1 to −2.3 µm and a total dose of 50.81 e^−^ Å^−2^ over 50 frames and an exposure time of 2.2 s (Supplementary Table S2).

All data processing was completed in *CryoSPARC* (Punjani *et al.*, 2017 ▸), as summarized in Supplementary Fig. S3. The micrograph movies were patch motion corrected, patch CTF estimated and then curated to 9567 micrographs. Particles were picked with the *Blob* and *Topaz* algorithms followed by the removal of duplicate particles and several rounds of 2D classification to remove ‘junk’ particles, resulting in 714 000 particles. *Ab initio* reconstruction using the remaining particles produced ‘junk’ and reasonable-looking initial references for heterogeneous refinement (one ‘junk’ reference and two reasonable references) to further curate the particles. 597 000 particles were selected for non-uniform refinement, resulting in a consensus map of the overall complex with an average resolution of 3.22 Å. To improve the map quality in the GPCR region and the β-lactamase-BLIPII region of the map, separate local refinements with masks over these regions resulted in density maps with improved local quality (final maps are summarized in Supplementary Fig. S4).

The initial model was generated from PDB entry 6ps7 (the X-ray structure of A_2A_R bound to ZM241385; Ishchenko *et al.*, 2019 ▸), PDB entry 1jtd (the X-ray structure of BLIPII; Lim *et al.*, 2001 ▸) and a predicted model of A_2A_R-β-lactamase_Bli_ (Chai Discovery Team, 2024 ▸). This initial model was rigid-body-fitted into the consensus map of the overall complex with *ChimeraX* (Goddard *et al.*, 2018 ▸), manually adjusted into this map using *Coot* (Emsley *et al.*, 2010 ▸) and then further fitted into the two focused maps. A composite map was generated in *Phenix* (Liebschner *et al.*, 2019 ▸) from the two focused maps to allow structure refinement of the entire complex at once. The initial model was refined into the composite map with several rounds of *Phenix* real-space refinement and manual adjustments in *Coot* (Emsley *et al.*, 2010 ▸). The final structure was validated in *Phenix* (Liebschner *et al.*, 2019 ▸) and the ligand geometries were validated with *Mogul* as implemented by Global Phasing Ltd (Bruno *et al.*, 2004 ▸; Bricogne *et al.*, 2017 ▸). R.m.s.d. calculations were performed in *PyMOL* (Schrödinger).

## 3. Results

### 3.1. Selection of fusion tags and design of A_2A_R fusion constructs

In our approach, we focused on incorporating fusion tags into the ICL3 of GPCRs, a loop which has historically accommodated fusions to enable structure determination. To identify suitable proteins or protein domains for use as a fusion tag, we applied a multi-parameter selection strategy focused on mass, rigidity and steric compatibility. These parameters are important to ensure successful incorporation of the protein tag into ICL3 and to enable cryo-EM structure determination. The size of the fusion tag is a key factor because the insertion of a relatively small protein is likely to be better tolerated by GPCRs. However, since this fusion tag needs to function as a fiducial marker to facilitate particle alignment during cryo-EM data processing, it needs to add sufficient mass to a ∼45 kDa GPCR. One way to balance the insertion of a small fusion tag while producing a protein particle with a sufficiently large mass is through complexation with other proteins; therefore, fusion candidates with high-affinity protein binding partners were of high interest (*i.e.* Fusions 5–8 in Supplementary Table S1). Another key requirement is ensuring rigidity between the fiducial marker and the target protein. This is because the entire protein mass is employed to drive data alignment, and flexibility between these two domains would be detrimental to map resolution. We set out to form a double-helical connection between the fusion tag and TM5 and TM6 of the GPCR, therefore replacing ICL3 with a rigidly integrated fusion tag. Candidate fusion tags needed to have appropriately positioned N- and C- terminal α-helices to accomplish this. Finally, upon fusion the candidate tags not only needed to avoid steric clashes with the GPCR and the membrane/micelle but should also avoid distorting the antagonist-bound GPCR conformation.

We started by mining the PDB for proteins that met our set of specifications. After *in silico* and further manual curation, as detailed in Section 2, 15 candidates were shortlisted as potential fusion tags. These proteins were then evaluated by creating fusion constructs with A_2A_R, a well characterized class A GPCR, and *in silico* modelling with *AlphaFold*2 (Jumper *et al.*, 2021 ▸). The fusion constructs were designed by inserting the candidate tag into A_2A_R with a matrix of five exit and five entry points flanking the ICL3. The *AlphaFold*2-predicted models for each construct were assessed for helical continuity, confidence scores across the junction and preservation of the inactive receptor conformation. Based on these analyses, one fusion construct was chosen per tag, resulting in the selection of eight A_2A_R fusion constructs for experimental validation (Supplementary Tables S1 and S3).

### 3.2. Assessment of expression and aggregation of A_2A_R fusion proteins

**Figure 1:**
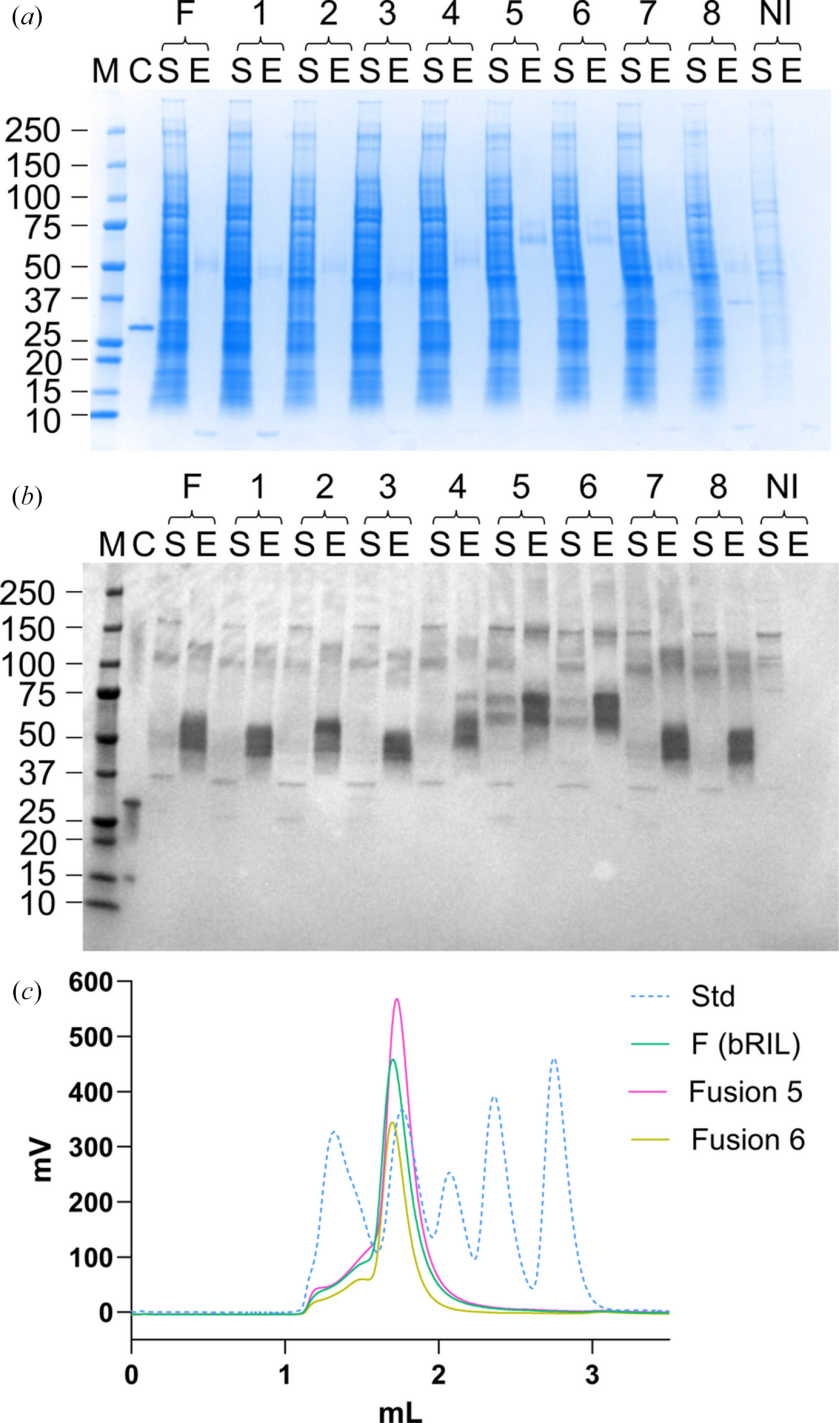
Assessment of A_2A_R with different fusion tags inserted into ICL3 from small-scale affinity pull-down experiments. (*a*) SDS–PAGE and (*b*) Western blot analysis of solubilized (S) and affinity pull-down elution (E) samples of A_2A_R fusion constructs (numbers correspond to the fusion constructs listed in Supplementary Table S1). M, molecular-weight standards in kDa; C, Western blot anti-FLAG control; NI, non-infected cell control; F, A_2A_R-bRIL fusion control. (*c*) fSEC analysis of A_2A_R with two β-lactamase fusions (Fusion 5, β-lactamase_Bli_, and Fusion 6, β-lactamase_Ban_) inserted into ICL3, with A_2A_R-bRIL fusion control [F (bRIL)] and molecular-weight standards (Std), at 670, 158, 44, 17 and 1.35 kDa. fSEC readings were collected with an excitation wavelength of 488 nm and an emission wavelength of 512 nm; the molecular-weight standards readings (Std) are from the *A*_280_ signal.

A_2A_R with eight different tags fused to TM5 and TM6 or a control fusion tag (bRIL) was expressed in small-scale insect-cell cultures, detergent-solubilized and the protein was purified by affinity pull-down for characterization. SDS–PAGE and Western blot analysis showed detectable levels of protein after affinity elution for all constructs (Fig. 1 ▸; A_2A_R-fusion protein is visible between the 37 and 75 kDa markers as double bands on the Western blot in Fig. 1 ▸*b*). The appearance of A_2A_R as a double band, especially evident in the Western blot, is explained by multiple glycosylation states of Asn154 (Jaakola *et al.*, 2008 ▸). The aggregation state of these A_2A_R constructs was evaluated by fluorescence size-exclusion chromatography (fSEC; Fig. 1 ▸, Supplementary Fig. S1). The fSEC profiles show clear peaks at ∼170 kDa for all eight fusion constructs, which matches a monomeric A_2A_R fusion embedded in a detergent micelle. Based on these data, we selected two constructs for further characterization by SPR. Fusion constructs 5–8 were initially shortlisted since they have known protein binders which would increase the mass of these fiducial markers without requiring Fab generation or purification. Of these four fusion tags, A_2A_R-Fusion 8 displays the lowest signal for the monomeric fSEC peak (Supplementary Fig. S1, peak height of less than 300 mV for construct 8 compared with constructs 5, 6 and 7). When comparing the binding partners for fusion tags 5 and 6 (β-lactamase inhibitory protein II; BLIPII) and fusion tag 7 (pectin methyl transferase; PME), PME is reportedly purified directly from the source (tomato fruit; Di Matteo *et al.*, 2005 ▸), whereas BLIPII is recombinantly expressed and purified from a scalable bacterial system (Brown *et al.*, 2011 ▸). This analysis, taken together with the previous *AlphaFold*2 prediction analysis, led to the selection of A_2A_R-β-lactamase_Bli_ (Fusion 5) and A_2A_R-β-lactamase_Ban_ (Fusion 6) for further characterization.

Compared with A_2A_R-Fusion 6 (β-lactamase_Ban_ from *Bacillus anthracis*), A_2A_R-Fusion 5 (β-lactamase_Bli_ from *B. licheniformis*) produced a higher yield of monomeric protein, as indicated by a greater fSEC peak signal height (Fig. 1 ▸*c*). This suggests the β-lactamase from *B. licheniformis*, a mesophilic bacterium, may form a more stable chimeric protein compared with the β-lactamase from *B. anthracis*.

### 3.3. Confirmation that A_2A_R with β-lactamase fusions bind ligands

**Figure 2:**
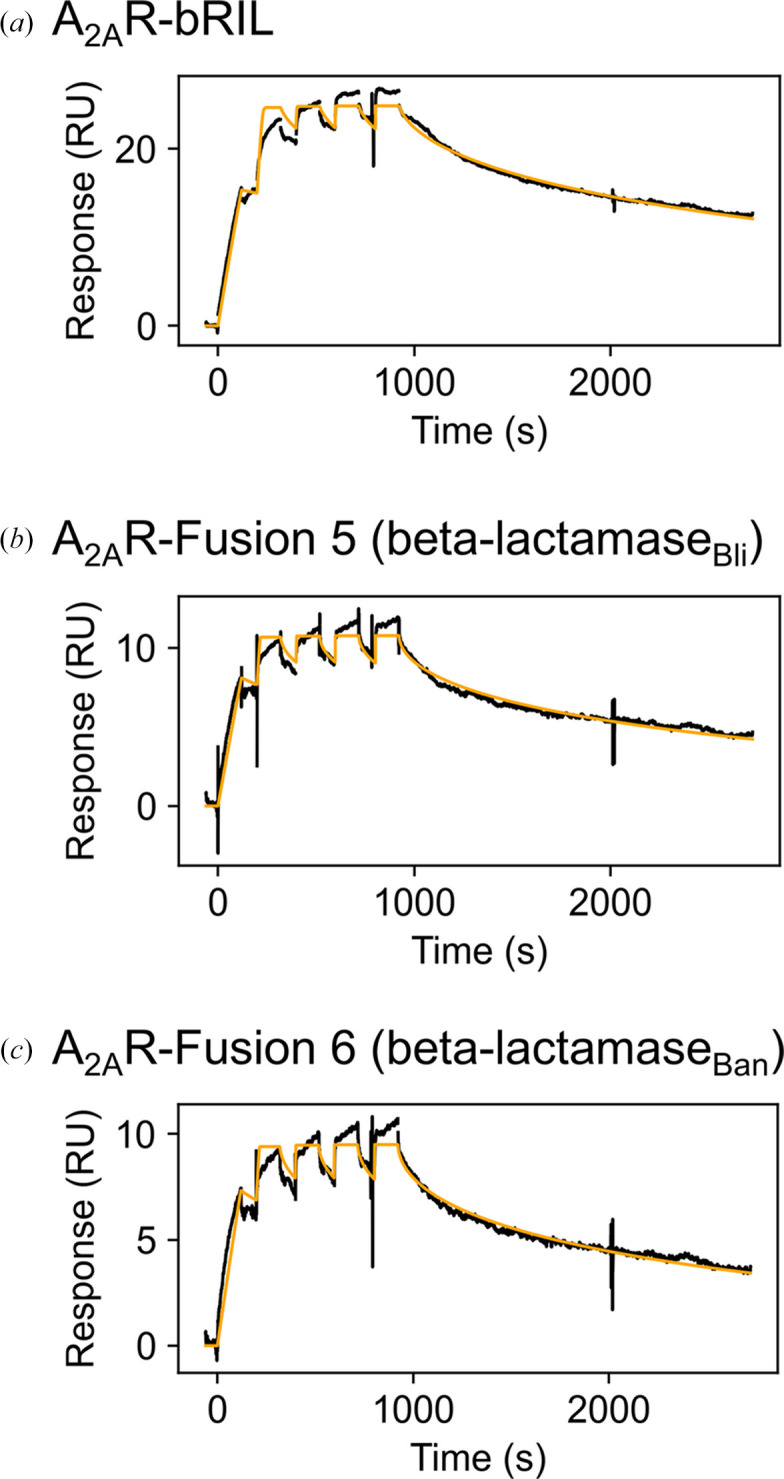
Binding sensorgram for antagonist ligand (ZM241385) binding to A_2A_R with (*a*) bRIL fusion, (*b*) Fusion 5 (β-lactamase_Bli_) and (*c*) Fusion 6 (β-lactamase_Ban_). ZM241385 was injected in single-cycle kinetics mode in a five-point concentration series ranging from 1 µ*M* to 12 n*M* with threefold dilution. Black lines represent experimental data and orange lines represent a 1:1 kinetic fit. RU, response units.

To further validate our constructs, we confirmed that the addition of the fusion did not disrupt ligand binding to A_2A_R. Both A_2A_R-β-lactamase proteins (Fusion 5 and Fusion 6) and a control, A_2A_R-bRIL, were purified in the absence of ligand for binding characterization by SPR. Comparison of the β-lactamase fusion constructs with the control construct, A_2A_R-bRIL, showed comparable kinetic profiles and binding affinities within a fivefold difference for KW3902 and LUF5834 and within a threefold difference for ZM241385, ANR94 and PSB0777. These differences are not considered significant as they are within the expected range of error for small molecule–protein interactions using Biacore technology, especially considering singlicate experiments (Cannon *et al.*, 2004 ▸; Papalia *et al.*, 2006 ▸). These results are also in line with reported binding affinities and *K*_i_ for A_2A_R. This was consistent across different ligand modalities, including antagonist compounds (ZM241385, ANR94, KW3902), a full agonist (PSB0777) and a partial agonist (LUF5834) (Table 1 ▸, Fig. 2 ▸, Supplementary Fig. S2; Segala *et al.*, 2015 ▸; Davalli *et al.*, 2025 ▸; El-Tayeb *et al.*, 2011 ▸; Beukers *et al.*, 2004 ▸). We therefore concluded that the insertion of β-lactamases into ICL3 does not impair the ability of A_2A_R to bind different ligands and does not appear to bias the receptor towards a more antagonist- or agonist-binding competent state.

### 3.4. Structure determination of antagonist-bound A_2A_R with β-lactamase_Bli_ and BLIPII

**Figure 3:**
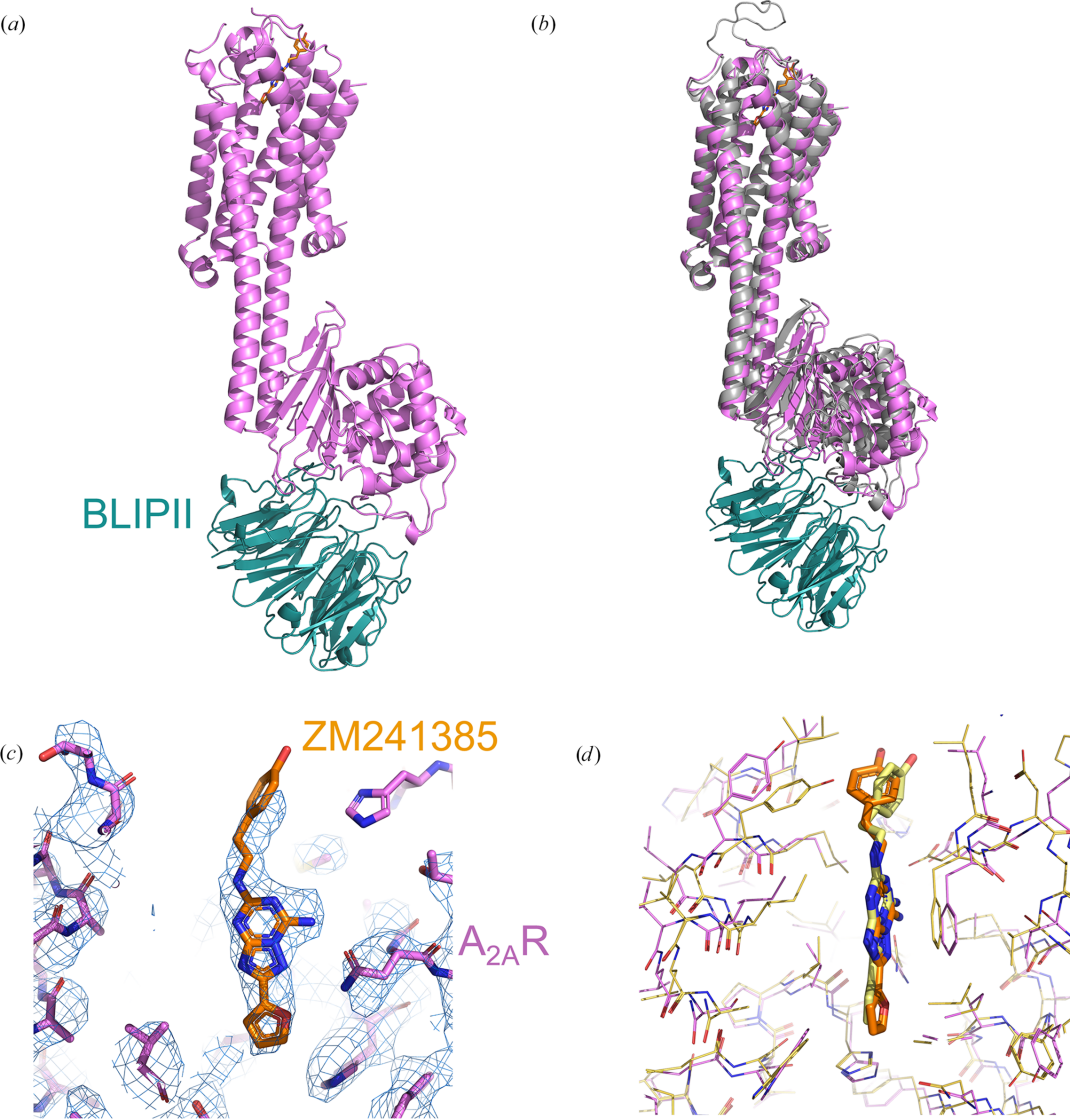
Cryo-EM structure of A_2A_R-β-lactamase_Bli_ (Fusion 5) bound to BLIPII and antagonist ligand ZM241385. (*a*) Overall structural model: A_2A_R-β-lactamase_Bli_ is in pink, BLIPII is in teal and ZM241385 is in orange. (*b*) Overlay of the cryo-EM experimental structure (pink and teal) with the *AlphaFold*2-predicted structure (grey), aligned on A_2A_R. Overlay of the full model (not shown) has an r.m.s.d. of 2.09 Å. (*c*) Ligand-binding site of the cryo-EM structure with the density map in blue, showing the binding pose of ZM241385 (orange). The map is displayed at 14σ. (*d*) Comparison of the ligand-binding site between the cryo-EM structure (pink and orange) and X-ray crystallographic structure of ZM241385-bound A_2A_R (PDB entry 5iu4, yellow).

A_2A_R fused to the *B. licheniformis* β-lactamase (A_2A_R-β-lactamase_Bli_) was purified in complex with β-lactamase inhibitory protein II (BLIPII) and small-molecule antagonist ZM241385 to assess whether this new ‘bulking-up’ tool could lead to a high-resolution structure of A_2A_R. The overall map and structure of A_2A_R-β-lactamase_Bli_–ZM241385 shows continuous helices between A_2A_R and β-lactamase_Bli_ (Fig. 3 ▸*a*, Supplementary Fig. S4*a*) and is remarkably similar to the *AlphaFold*2-predicted model, with an r.m.s.d. of 2.09 Å (Fig. 3 ▸*b*). The overall mass and rigidity between these two domains were sufficient to drive reliable angular assignment during cryo-EM data processing, leading to an average resolution of 3.22 Å across the full complex (Supplementary Fig. S4*a*). Analysis of the structure supports the rationally engineered R72S mutation in the β-lactamase domain. In the junction region between A_2A_R and β-lactamase_Bli_, Arg72 of the β-lactamase was identified as a potential source of steric clash with Gln266 of A_2A_R. Comparing the experimental structure with the predicted model highlights how this serine avoids a potential clash in the helical connection region (Supplementary Fig. S5).

The orthosteric ligand-binding site for ZM241385 is situated at the extracellular edge of A_2A_R and this region is not well resolved in the density map of the full complex. A local refinement focused around A_2A_R improved the local resolution and map features in this region (Supplementary Fig. S4), thereby allowing confident placement of the central bicyclic moiety and furan ring of ZM241385 (Fig. 3 ▸*c*). In the current cryo-EM structure, extracellular loop 2 (ECL2) of A_2A_R is not well defined, which suggests that it is flexible in solution (Fig. 3 ▸*a*, Supplementary Fig. S6). In contrast, the X-ray crystallographic structure of A_2A_R bound to ZM241385 (PDB entry 5iu4; Segala *et al.*, 2016 ▸) has a modelled ECL2 since the bRIL fusion of a symmetry-related A_2A_R-bRIL packs against and stabilizes the conformation of this loop. The pose of ZM241385 overlaps well between these structures (Fig. 3 ▸*d*) with one noticeable difference in the angle of the phenol ring. The phenol ring of ZM241385 in the crystal structure is better resolved, therefore justifying the position, whereas the density for the phenol ring in the current cryo-EM structure is not as well defined (Fig. 3 ▸*c*), which led to the refinement of ZM241385 with more idealized ligand geometry in this region.

## 4. Conclusions and discussion

We have demonstrated that fusing β-lactamase_Bli_ into ICL3 and complexation with BLIPII, thereby adding ∼57 kDa to a GPCR, is a viable approach for structure solution of antagonist-bound GPCRs by cryo-EM.

In the literature, the bRIL fusion method (combined with an anti-bRIL Fab and anti-Fab VHH) is prevalent for solving antagonist-bound GPCR structures (Zhang *et al.*, 2022 ▸). In this system the bRIL moiety, which is connected to TM5 and TM6 with continuous helices, often sits ‘underneath’ the intracellular region of the GPCR. This can lead to possible clashes between the bRIL fusion and intracellular GPCR loops, depending on the TM helix and ICL sizes and architectures. Furthermore, depending on the angle of the bRIL in relation to the GPCR and detergent micelle, the anti-bRIL Fab can be an additional source of clashes (for example with the detergent micelle). Compared with this approach, the rigidly connected β-lactamase does not sit underneath the GPCR but rather points outwards towards the solvent and away from the cytoplasmic surface of the GPCR and ICLs (Supplementary Fig. S6). BLIPII binds to the β-lactamase with a downward angle pointing away from the GPCR, and collectively the β-lactamase-BLIPII fusion complex is better positioned to minimize the risk of steric hinderance (Fig. 3 ▸, Supplementary Fig. S6). In this study, the fSEC profile of A_2A_R-β-lactamase_Bli_ after affinity pull-down shows a better monomer peak height compared with the A_2A_R-bRIL construct, suggesting that the β-lactamase fusion generates a more stable protein (Fig. 1 ▸*c*).

In a preprint deposited on *bioRxiv*, Collu and coworkers describe a similar attempt to establish a β-lactamase, AmpC, as a GPCR fusion tool. This study describes resolving the seven-TM-helical architecture of β_1_-adrenoceptor (β_1_AR) by cryo-EM using a β-lactamase fusion in ICL3, but the ligand-binding pose was not determined due to limited map resolution and quality (Collu *et al.*, 2021 ▸). Two possible reasons for the challenges faced by Collu and coworkers are (i) the connection between the β-lactamase fusion and β_1_AR is too flexible and/or (ii) the β-lactamase alone is not of sufficient size to drive high-resolution cryo-EM data alignment. We have overcome the first potential obstacle by utilizing *AlphaFold*2 (Jumper *et al.*, 2021 ▸) to efficiently assess engineered fusion connections for rigidity and select the most promising constructs. Regarding the second point, the potential size limitation of a β-lactamase fusion, we created a larger fiducial marker by complexation with BLIPII. The approach in the current study has enabled us to resolve the ligand-binding pocket to ∼3–3.2 Å.

As the field continues to generate new methods to solve GPCR structures, such as the RF diffusion-generated Clip1 and Clip2 fusion tags (Gao *et al.*, 2025 ▸) and a new fusion strategy involving heterodimeric calcineurin protein (Xu *et al.*, 2024 ▸), β-lactamase with the BLIPII binding partner adds to the portfolio of available fusion tools to facilitate GPCR structural biology and structure-based drug design.

## 5. Related literature

The following references are cited in the supporting information for this article: Anandapadamanaban *et al.* (2019 ▸), Fonzé *et al.* (2002 ▸), Kozbial *et al.* (2008 ▸), Moews *et al.* (1990 ▸), Pain (2014 ▸), Quezada *et al.* (2004 ▸) and Zheng *et al.* (2009 ▸).

## Supplementary Material

PDB reference: A_2A_R-β-lactamase_Bli_ bound to ZM241385, 9t9p

EMDB reference: A_2A_R-β-lactamase_Bli_ bound to ZM241385, composite map, EMD-55723

EMDB reference: overall consensus map, EMD-56449

EMDB reference: β-lactamase- and BLIPII-focused map, EMD-56450

EMDB reference: A_2A_R-focused map, EMD-56451

Supplementary Tables and Figures. DOI: 10.1107/S2059798326003785/vo5023sup1.pdf

## Figures and Tables

**Table 1:** Ligand binding to A_2A_R with different fusion tags inserted into ICL3, measured by SPR, compared with literature-reported binding to A_2A_R

Ligand	Ligand-binding mode	A_2A_R-bRIL *K*_d_ (n*M*) (this study)	A_2A_R-Fusion 5 *K*_d_ (n*M*) (β-lactamase_Bli_)	A_2A_R-Fusion 6 *K*_d_ (n*M*) (β-lactamase_Ban_)	A_2A_StaR2 in DDM micelles *K*_d_ (n*M*)	A_2A_R *K*_i_ (n*M*)
ZM241385	Antagonist	0.34	0.38	0.57	0.39 (Segala *et al.*, 2015 ▸)	0.59 (Segala *et al.*, 2015 ▸)
ANR94	Antagonist	130	71	92		46 (Pinna *et al.*, 2005 ▸)
KW3902	Antagonist	150	104	29.3	33 (Segala *et al.*, 2015 ▸)	34 (Segala *et al.*, 2015 ▸)
PSB0777	Agonist	66.9	108	143		219 (Navarro *et al.*, 2020 ▸)
LUF5834	Partial agonist	12.9	50.9	57.2		13 (Navarro *et al.*, 2020 ▸)

## Data Availability

The structure and maps of A_2A_R-β-lactamase_Bli_ bound to ZM241385 have been deposited as PDB entry 9t9p and EMDB entries EMD-55723 (composite map), EMD-56449 (overall consensus map), EMD-56450 (β-lactamase and BLIPII-focused map) and EMD-56451 (A_2A_R-focused map).
