# Supplementary material for: A novel fusion tool to enable G protein-coupled receptor structure determination
Source: Acta Crystallogr D Struct Biol. 2026 May 15;82(Pt 6):655–63. doi: 10.1107/S2059798326003785 (PMC13224925; doi:10.1107/S2059798326003785)
Supplement: Supplementary file 1 [file d-82-00655-sup1.pdf]

STRUCTURAL
BIOLOGY

Volume 82 (2026)

Supporting information for article:

A novel fusion tool to enable G protein-coupled receptor structure determination

Nita R. Shah, Mathieu Oosterlaken, Claudine Bisson, Mattia Bertinelli, Alicia M. Churchill-Angus, Andrew Hutchin, Jola Kopec, Vadim Kotov, Ciaran R. McFarlane, Erika Griss Pascualli, Ana Pavic, Matthias Zebisch, Cédric Fiez-Vandal, Edoardo Fabini and Stéphanie Duclos

Table S1 List of $A_{2A}R$ constructs with fusion tags inserted into ICL3

Fusion construct number	Fusion tag identity (UniProt ID)	Source organism	Fusion tag residue range*	Fusion point in $A_{2A}R$*+	Point mutations*	Reference PDB
1	CheA phosphotransfe rase domain (Q56310)	*Thermotoga maritima*		M211, A221	$A_{2A}R$: M211L<br>Fusion: G7S, G49R, G52E, M94E	1TQG (Quezada *et al.*, 2004)
2	Lin2004 protein (Q92AB8)	*Listeria innocua*	E2-R117	E212, R222	$A_{2A}R$: M211L<br>Fusion: F33K, Y34E, C47S, M81E, F92Y	2HUJ (Kozbial *et al.*, 2008)
3	APC36109 (Q5KVS1)	*Geobacillus kaustophilus*	N7-A82	L208, Q226	$A_{2A}R$: none<br>Fusion: A81T, A82V, H70D	1U84
4	TM0439 (Q9WYS0)	*Thermotoga maritima*	K77-N207	E212, A221	$A_{2A}R$: M211L<br>Fusion: M88K, G112N, L145Q, L149E	3SXZ (Zheng *et al.*, 2009)
5	Beta-lactamase (P00808)	*Bacillus licheniformis*	M46-L302	Q214, R220	$A_{2A}R$: none<br>Fusion: M46A, R72S, A301L	4M3K (Pain, 2014), 2BLM (Moews *et al.*, 1990)**
6	Beta-lactamase (Q93T42)	*Bacillus anthracis*	T52-F308	Q214, R220	$A_{2A}R$: none<br>Fusion: T52V	3QHY (Brown *et al.*, 2011)
7	Pectinesterase inhibitor (P83326)	*Actinidia chinensis*	K66-P182	M211, R220	$A_{2A}R$: R222A<br>Fusion: G67S, L68Q, G69A, F71K, I75E, G101A, G118N, I173R, L180E, P182A	1XG2 (Di Matteo *et al.*, 2005)

8	RagA insertion (Q7L523) co-expressed with RagC (Q9HB90)	Homo sapiens	Q185-R302<br>RagC*** S59-S376	E212, A221	A_{2A}R: None<br>RagA: C219S<br>RagC: S75N, C297S, C358S	6S6D (Anandapadamanaban *et al.*, 2019)

* Residue numbering according to UniProt sequence entry

** PDB 2BLM matches the UniProt entry and construct sequence used in this study, PDB 4M3K was the initial structure used for tag assessment and connectivity analysis, but it has an extra 2 residues compared to the P00808 UniProt entry (Pro-Gly inserted between D211 and K212)

*** RagC was co-expressed with an N-terminal 6xHis-tag.

\+ Fusion point denotes the residues that are still part of the construct (the insertion is after the first residue and before the second residue)

Table S2 Data collection, processing, and refinement statistics for cryo-EM structure of A_{2A}R-beta-lactamase$_{Bli}$ + BLIPII + ZM241385.

Data collection and processing	Magnification	105k
	Voltage (kV)	300
	Electron exposure (e Å^2)	50.81
	Defocus range (μm)	-1.1 to -2.3
	Pixel size (Å)	0.835
	Symmetry imposed	C1
	No. of initial particle images	Blob picking = 2.9 million<br>Topaz = 1.4 million
	No. of final particle images	597k
	Map resolution FSC threshold (Å) (threshold = 0.143)	A_{2A}R focused map: 3.43<br>Beta-lactamase + BLIPII focused map: 2.77
	Map resolution range (Å)	A_{2A}R focused map: ~3.0 to 4.0<br>Beta-lactamase + BLIPII focused map: ~2.3 to 3.0
Refinement	Initial model used	PDB 6PS7 (Ishchenko *et al.*, 2019), PDB 1JTD (Lim *et al.*, 2001), Chai prediction of

	A_{2A}R-beta-lactamase$_{Bli}$
Model resolution FSC threshold (Å)	0.143
Map sharpening *B* factor (Å^2)	A_{2A}R focused map: -143
	Beta-lactamase + BLIPII focused map: -90.6
Model composition	
Non-hydrogen atoms	6116
Protein/nucleic acid	6091
Ligand	25
R.m.s. deviations from ideality	Restraints from PHENIX 1.20.1_4487 (Liebschner *et al.*, 2019)
Bonds (Å)	0.002
Angles (º)	0.459
Mol probity score	1.38
Clash score	4.25
Poor rotamers (%)	0
Ramachandran plot	Restraints from PHENIX 1.20.1_4487 (Liebschner *et al.*, 2019)
Favoured (%)	96.99
Allowed (%)	3.01
Disallowed (%)	0

Composite map (EMDB-55723) generated from the A_{2A}R-focused map (EMD-56451) and beta-lactamase+BLIPII-focused map (EMDB-56450) was used for refinement. Final model statistics for PDB 9T9P.

Table S3 Sequence of $A_{2A}R$ constructs with fusion tags inserted into ICL3

$A_{2A}R$-Fusion construct number	Protein sequence
1	MKTIIALSYIFCLVFADYKDDDDKPIMGSSVYITVELAIAVLAILGNVLVCWAVWLNSNL<br>QNVTNYFVVSLAAADIAVGVLAIPFAITISTGFCAACHGCLFIACFVLVLTQSSIFSLLAIAI<br>DRYIAIRIPLRYNGLVTGTRAKGIIAICWVLSFAIGLTPMLGWNNCGQPKEGKNHSQGCG<br>EGQVACLFEDVVPMNYMVYFNFFACVLVPLLLMLGVYLRIFLAARRQLKQLSVFVDET<br>KEYLQNLNDTLLELEKNPEDMELINEAFRALHTLKRMAETMGFSSMAKLCHTLENILDK<br>ARNSEIKITSDLLDKIFAGVDEITRMVDKIVSARSTLQKEVHAAKSLAIIVGLFALCWLPL<br>HIINCFTFFCPDCSHAPLWLMYLAIVLSHTNSVVNPFIYAYRIREFRQTFRKIIRSHVLRQQ<br>EPFKAENLYFQGVSKGEELFTGVVPILVELDGDVNGHKFSVSGEGEGDATYGKLTLKFIC<br>TTGKLPVPWPTLVTTLTYGVQCFSRYPDHMKQHDFFKSAMPEGYVQERTIFFKDDGNYK<br>TRAEVKFEGDTLVNRIELKGIDFKEDGNILGHKLEYNYNSHNVYIMADKQKNGIKVNFKI<br>RHNIEDGSVQLADHYQQNTPIGDGPVLLPDNHYLSTQSKLSKDPNEKRDHMVLLEFVTA<br>AGITLGMDELYKHHHHHHHHHH
2	MKTIIALSYIFCLVFADYKDDDDKPIMGSSVYITVELAIAVLAILGNVLVCWAVWLNSNL<br>QNVTNYFVVSLAAADIAVGVLAIPFAITISTGFCAACHGCLFIACFVLVLTQSSIFSLLAIAI<br>DRYIAIRIPLRYNGLVTGTRAKGIIAICWVLSFAIGLTPMLGWNNCGQPKEGKNHSQGCG<br>EGQVACLFEDVVPMNYMVYFNFFACVLVPLLLMLGVYLRIFLAARRQLKQLEELLIRTE<br>QLLLQNEKNWELYLSNREEEKPFDKEKDMKPFVDEAKRSADDFLELAIPWVNTERPPYL<br>GELQLRQACDNVQETAVSAFNGRSYYKHFLDHYQSTKYTLTRVRDFLKRKRSTLQKEV<br>HAAKSLAIIVGLFALCWLPLHIINCFTFFCPDCSHAPLWLMYLAIVLSHTNSVVNPFIYAY<br>RIREFRQTFRKIIRSHVLRQQEPFKAENLYFQGVSKGEELFTGVVPILVELDGDVNGHKFS<br>VSGEGEGDATYGKLTLKFICTTGKLPVPWPTLVTTLTYGVQCFSRYPDHMKQHDFFKSA<br>MPEGYVQERTIFFKDDGNYKTRAEVKFEGDTLVNRIELKGIDFKEDGNILGHKLEYNYNS<br>HNVYIMADKQKNGIKVNFKIRHNIEDGSVQLADHYQQNTPIGDGPVLLPDNHYLSTQSK<br>LSKDPNEKRDHMVLLEFVTAAGITLGMDELYKHHHHHHHHHH
3	MKTIIALSYIFCLVFADYKDDDDKPIMGSSVYITVELAIAVLAILGNVLVCWAVWLNSNL<br>QNVTNYFVVSLAAADIAVGVLAIPFAITISTGFCAACHGCLFIACFVLVLTQSSIFSLLAIAI<br>DRYIAIRIPLRYNGLVTGTRAKGIIAICWVLSFAIGLTPMLGWNNCGQPKEGKNHSQGCG<br>EGQVACLFEDVVPMNYMVYFNFFACVLVPLLLMLGVYLRIFLAARRQLNRLLLEWIGA<br>WDPFGLGKDAYDVEAASVLQAVYETEDARTLAARIQSIYEFAFDEPIPFPHCLKLARRLL<br>ELKQTVQKEVHAAKSLAIIVGLFALCWLPLHIINCFTFFCPDCSHAPLWLMYLAIVLSHTN<br>SVVNPFIYAYRIREFRQTFRKIIRSHVLRQQEPFKAENLYFQGVSKGEELFTGVVPILVELD<br>GDVNGHKFSVSGEGEGDATYGKLTLKFICTTGKLPVPWPTLVTTLTYGVQCFSRYPDHM<br>KQHDFFKSAMPEGYVQERTIFFKDDGNYKTRAEVKFEGDTLVNRIELKGIDFKEDGNILG

	HKLEYNYNSHNVYIMADKQKNGIKVNFKIRHNIEDGSVQLADHYQQNTPIGDGPVLLPD<br>NHYLSTQSKLSKDPNEKRDHMVLLEFVTAAGITLGMDELYKHHHHHHHHHH
4	MKTIIALSYIFCLVFADYKDDDDKPIMGSSVYITVELAIAVLAILGNVLVCWAVWLNSNL<br>QNVTNYFVVSLAAADIAVGVLAIPFAITISTGFCAACHGCLFIACFVLVLTQSSIFSLLAIAI<br>DRYIAIRIPLRYNGLVTGTRAKGIIAICWVLSFAIGLTPMLGWNNCGQPKEGKNHSQGCG<br>EGQVACLFEDVVPMNYMVYFNFFACVLVPLLLMLGVYLRIFLAARRQLKQLEKFIRETIE<br>TRIKMEVFCLENYFDKIAGSEELLEIKNEIDDVEKSAKREIFDDSDERLHKLFIRASGNEQII<br>SEYEKIWDRIDLVRHLNERYVVSNREHKELIERIISGDKEGAIEKLKEHLKNVEAETIKNA<br>RSTLQKEVHAAKSLAIIVGLFALCWLPLHIINCFTFFCPDCSHAPLWLMYLAIVLSHTNSV<br>VNPFIYAYRIREFRQTFRKIIRSHVLRQQEPFKAENLYFQGVSKGEELFTGVVPILVELDGD<br>VNGHKFSVSGEGEGDATYGKLTLKFICTTGKLPVPWPTLVTTLTYGVQCFSRYPDHMKQ<br>HDFFKSAMPEGYVQERTIFFKDDGNYKTRAEVKFEGDTLVNRIELKGIDFKEDGNILGHK<br>LEYNYNSHNVYIMADKQKNGIKVNFKIRHNIEDGSVQLADHYQQNTPIGDGPVLLPDNH<br>YLSTQSKLSKDPNEKRDHMVLLEFVTAAGITLGMDELYKHHHHHHHHHH
5	MKTIIALSYIFCLVFADYKDDDDKPIMGSSVYITVELAIAVLAILGNVLVCWAVWLNSNL<br>QNVTNYFVVSLAAADIAVGVLAIPFAITISTGFCAACHGCLFIACFVLVLTQSSIFSLLAIAI<br>DRYIAIRIPLRYNGLVTGTRAKGIIAICWVLSFAIGLTPMLGWNNCGQPKEGKNHSQGCG<br>EGQVACLFEDVVPMNYMVYFNFFACVLVPLLLMLGVYLRIFLAARKRIKRLTEFIEKSEK<br>LLKEAEAALKQLKKDPNNKELIEKILKLYKELEKLAKEEGYEEVAKLAKEIAKVFEKILK<br>GEEKLTPELLKKIEAGLKKIKAELEKEKERLERLRREVHAAKSLAIIVGLFALCWLPLHIIN<br>CFTFFCPDCSHAPLWLMYLAIVLSHTNSVVNPFIYAYRIREFRQTFRKIIRSHVLRQQEPFK<br>AENLYFQGVSKGEELFTGVVPILVELDGDVNGHKFSVSGEGEGDATYGKLTLKFICTTGK<br>LPVPWPTLVTTLTYGVQCFSRYPDHMKQHDFFKSAMPEGYVQERTIFFKDDGNYKTRAE<br>VKFEGDTLVNRIELKGIDFKEDGNILGHKLEYNYNSHNVYIMADKQKNGIKVNFKIRHNI<br>EDGSVQLADHYQQNTPIGDGPVLLPDNHYLSTQSKLSKDPNEKRDHMVLLEFVTAAGIT<br>LGMDELYKHHHHHHHHHH
6	MKTIIALSYIFCLVFADYKDDDDKPIMGSSVYITVELAIAVLAILGNVLVCWAVWLNSNL<br>QNVTNYFVVSLAAADIAVGVLAIPFAITISTGFCAACHGCLFIACFVLVLTQSSIFSLLAIAI<br>DRYIAIRIPLRYNGLVTGTRAKGIIAICWVLSFAIGLTPMLGWNNCGQPKEGKNHSQGCG<br>EGQVACLFEDVVPMNYMVYFNFFACVLVPLLLMLGVYLRIFLAARRQLKQMESQVHKE<br>FSQLEKKFDARLGVYAIDTGTNQTIAYRPNERFAFASTYKALAAGVLLQQNSTKKLDEVI<br>TYTKEDLVDYSPVTEKHVDTGMTLGEIAEAAVRYSDNTAGNILFHKIGGPKGYEKALRQ<br>MGDRVTMSDRFETELNEAIPGDIRDTSTAKAIATNLKAFTAGNALPNHKRNILTKWMKG<br>NATGDKLIRAGVPTNWVVADKSGAGSYGTRNDIAIVWPPNRAPIIIAILSSKDEKGATYD<br>NQLIAEAAEVIVNAFRARSTLQKEVHAAKSLAIIVGLFALCWLPLHIINCFTFFCPDCSHAP<br>LWLMYLAIVLSHTNSVVNPFIYAYRIREFRQTFRKIIRSHVLRQQEPFKAENLYFQGVSKG<br>EELFTGVVPILVELDGDVNGHKFSVSGEGEGDATYGKLTLKFICTTGKLPVPWPTLVTTL<br>TYGVQCFSRYPDHMKQHDFFKSAMPEGYVQERTIFFKDDGNYKTRAEVKFEGDTLVNRI<br>ELKGIDFKEDGNILGHKLEYNYNSHNVYIMADKQKNGIKVNFKIRHNIEDGSVQLADHY

	QQNTPIGDGPVLLPDNHYLSTQSKLSKDPNEKRDHMVLLEFVTAAGITLGMDELYKHHH<br>HHHHHHH
7	MKTIIALSYIFCLVFADYKDDDDKPIMGSSVYITVELAIAVLAILGNVLVCWAVWLNSNL<br>QNVTNYFVVSLAAADIAVGVLAIPFAITISTGFCAACHGCLFIACFVLVLTQSSIFSLLAIAI<br>DRYIAIRIPLRYNGLVTGTRAKGIIAICWVLSFAIGLTPMLGWNNCGQPKEGKNHSQGCG<br>EGQVACLFEDVVPMNYMVYFNFFACVLVPLLLMLGVYLRIFLAARRQLKQMKSQAQKS<br>IDEAQASAKQTSKIIASLTNQATDPKLKARYETCSENYADAIDSLNQAKQFLTSGDYNSL<br>NIYASAAFDGAGTCEDSFEGPPNIPTQLHQADLKLEDLCDRVLVISNELARAASTLQKEV<br>HAAKSLAIIVGLFALCWLPLHIINCFTFFCPDCSHAPLWLMYLAIVLSHTNSVVNPFIYAY<br>RIREFRQTFRKIIRSHVLRQQEPFKAENLYFQGVSKGEELFTGVVPILVELDGDVNGHKFS<br>VSGEGEGDATYGKLTLKFICTTGKLPVPWPTLVTTLTYGVQCFSRYPDHMKQHDFFKSA<br>MPEGYVQERTIFFKDDGNYKTRAEVKFEGDTLVNRIELKGIDFKEDGNILGHKLEYNYNS<br>HNVYIMADKQKNGIKVNFKIRHNIEDGSVQLADHYQQNTPIGDGPVLLPDNHYLSTQSK<br>LSKDPNEKRDHMVLLEFVTAAGITLGMDELYKHHHHHHHHHH
8	MKTIIALSYIFCLVFADYKDDDDKPIMGSSVYITVELAIAVLAILGNVLVCWAVWLNSNL<br>QNVTNYFVVSLAAADIAVGVLAIPFAITISTGFCAACHGCLFIACFVLVLTQSSIFSLLAIAI<br>DRYIAIRIPLRYNGLVTGTRAKGIIAICWVLSFAIGLTPMLGWNNCGQPKEGKNHSQGCG<br>EGQVACLFEDVVPMNYMVYFNFFACVLVPLLLMLGVYLRIFLAARRQLKQMEQQLEM<br>NLRNFAQIIEADEVLLFERATFLVISHYQSKEQRDVHRFEKISNIIKQFKLSCSKLAASFQS<br>MEVRNSNFAAFIDIFTSNTYVMVVMSDPSIPSAATLINIRNARKHFEKLERARSTLQKEVH<br>AAKSLAIIVGLFALCWLPLHIINCFTFFCPDCSHAPLWLMYLAIVLSHTNSVVNPFIYAYRI<br>REFRQTFRKIIRSHVLRQQEPFKAENLYFQGVSKGEELFTGVVPILVELDGDVNGHKFSVS<br>GEGEGDATYGKLTLKFICTTGKLPVPWPTLVTTLTYGVQCFSRYPDHMKQHDFFKSAMP<br>EGYVQERTIFFKDDGNYKTRAEVKFEGDTLVNRIELKGIDFKEDGNILGHKLEYNYNSHN<br>VYIMADKQKNGIKVNFKIRHNIEDGSVQLADHYQQNTPIGDGPVLLPDNHYLSTQSKLSK<br>DPNEKRDHMVLLEFVTAAGITLGMDELYKHHHHHHHHHH
bRIL control	MKTIIALSYIFCLVFADYKDDDDKPIMGSSVYITVELAIAVLAILGNVLVCWAVWLNSNL<br>QNVTNYFVVSLAAADIAVGVLAIPFAITISTGFCAACHGCLFIACFVLVLTQSSIFSLLAIAI<br>DRYIAIRIPLRYNGLVTGTRAKGIIAICWVLSFAIGLTPMLGWNNCGQPKEGKNHSQGCG<br>EGQVACLFEDVVPMNYMVYFNFFACVLVPLLLMLGVYLRIFLAARRQLADLEDNWETL<br>NDNLKVIEKADNAAQVKDALTKMRAAALDAQKATPPKLEDKSPDSPEMKDFRHGFDIL<br>VGQIDDALKLANEGKVKEAQAAAEQLKTTRNAYIQKYLERARSTLQKEVHAAKSLAIIV<br>GLFALCWLPLHIINCFTFFCPDCSHAPLWLMYLAIVLSHTNSVVNPFIYAYRIREFRQTFR<br>KIIRSHVLRQQEPFKAENLYFQGVSKGEELFTGVVPILVELDGDVNGHKFSVSGEGEGDA<br>TYGKLTLKFICTTGKLPVPWPTLVTTLTYGVQCFSRYPDHMKQHDFFKSAMPEGYVQER<br>TIFFKDDGNYKTRAEVKFEGDTLVNRIELKGIDFKEDGNILGHKLEYNYNSHNVYIMADK<br>QKNGIKVNFKIRHNIEDGSVQLADHYQQNTPIGDGPVLLPDNHYLSTQSKLSKDPNEKRD<br>HMVLLEFVTAAGITLGMDELYKHHHHHHHHHH

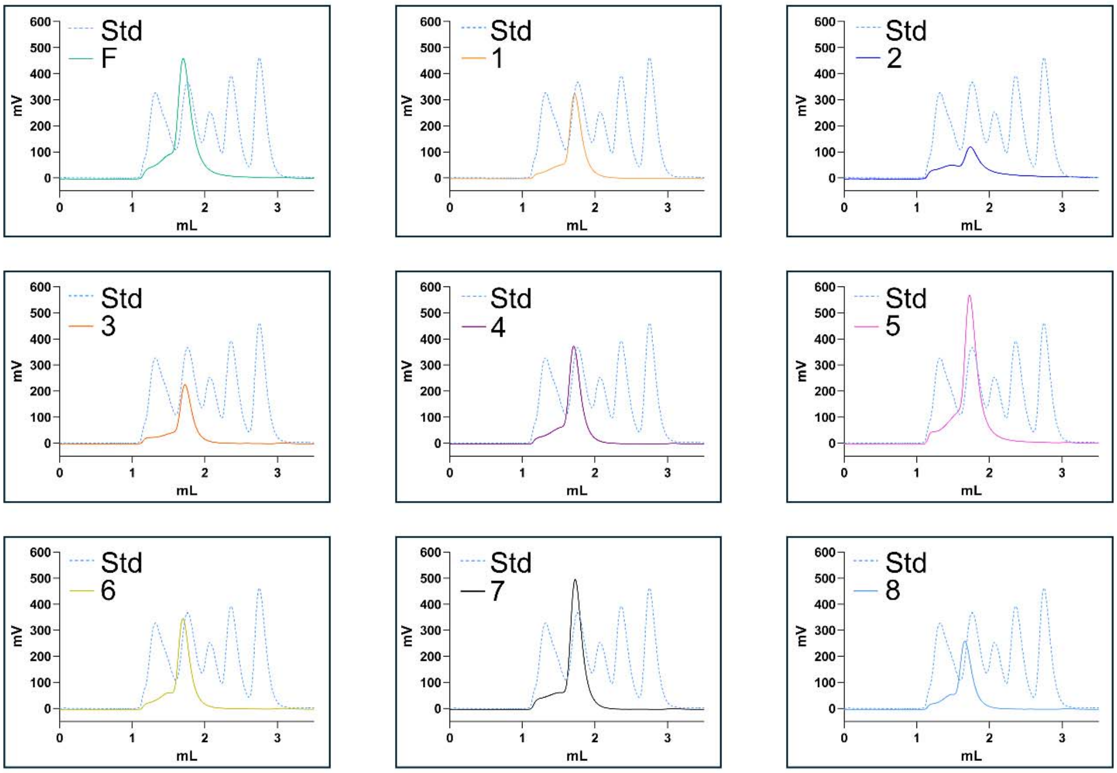

Figure S1 fSEC analysis of $A_{2A}R$ with different fusion tags inserted into ICL3. F = Fusion control (bRIL), numbers indicate the Fusion tag number (fusion constructs listed in Supplementary Table S1). Dashed blue line indicates molecular weight standards (Std), at: 670, 158, 44, 17, 1.35 kDa. fSEC readings collected with excitation wavelength of 488 nm and emission wavelength of 512 nm, the molecular weight standards curves (Std) are from A_{280} signal.

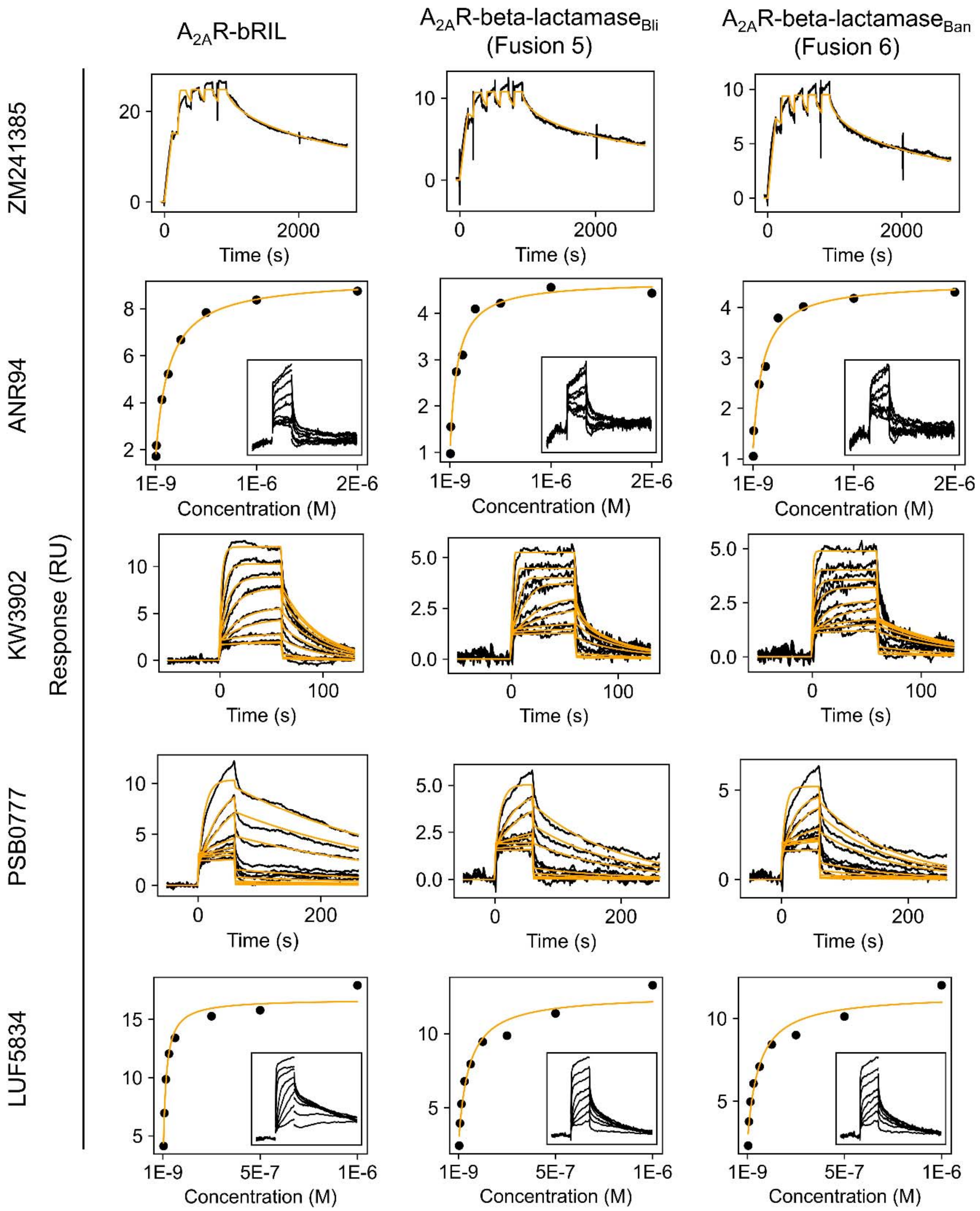

Figure S2 Ligand binding to $A_{2A}R$ with bRIL fusion, Fusion 5 (beta-lactamase$_{Bli}$), and Fusion 6 (beta-lactamase$_{Ban}$). ANR94, KW3902, PSB0777, LUF5834 were injected in multi-cycle kinetics mode in a ten-points concentration series ranging from 2 µM to 3.9 nM with two-fold dilution scheme. For LUF5834, 2µM concentration was excluded from the analysis due to unspecific binding. ZM241385 was injected in single-cycle kinetics mode in a five-points concentration series ranging from 1 µM to 12 nM with three-fold dilution. For ANR94 and LUF5834, affinity was determined

from steady-state plot (shown in the inset), for KW3902 and PSB0777, affinity was derived from kinetic fitting of the data to a simple 1:1 binding model. Black lines represent experimental data and orange lines represent 1:1 kinetic fit. RU (response units).

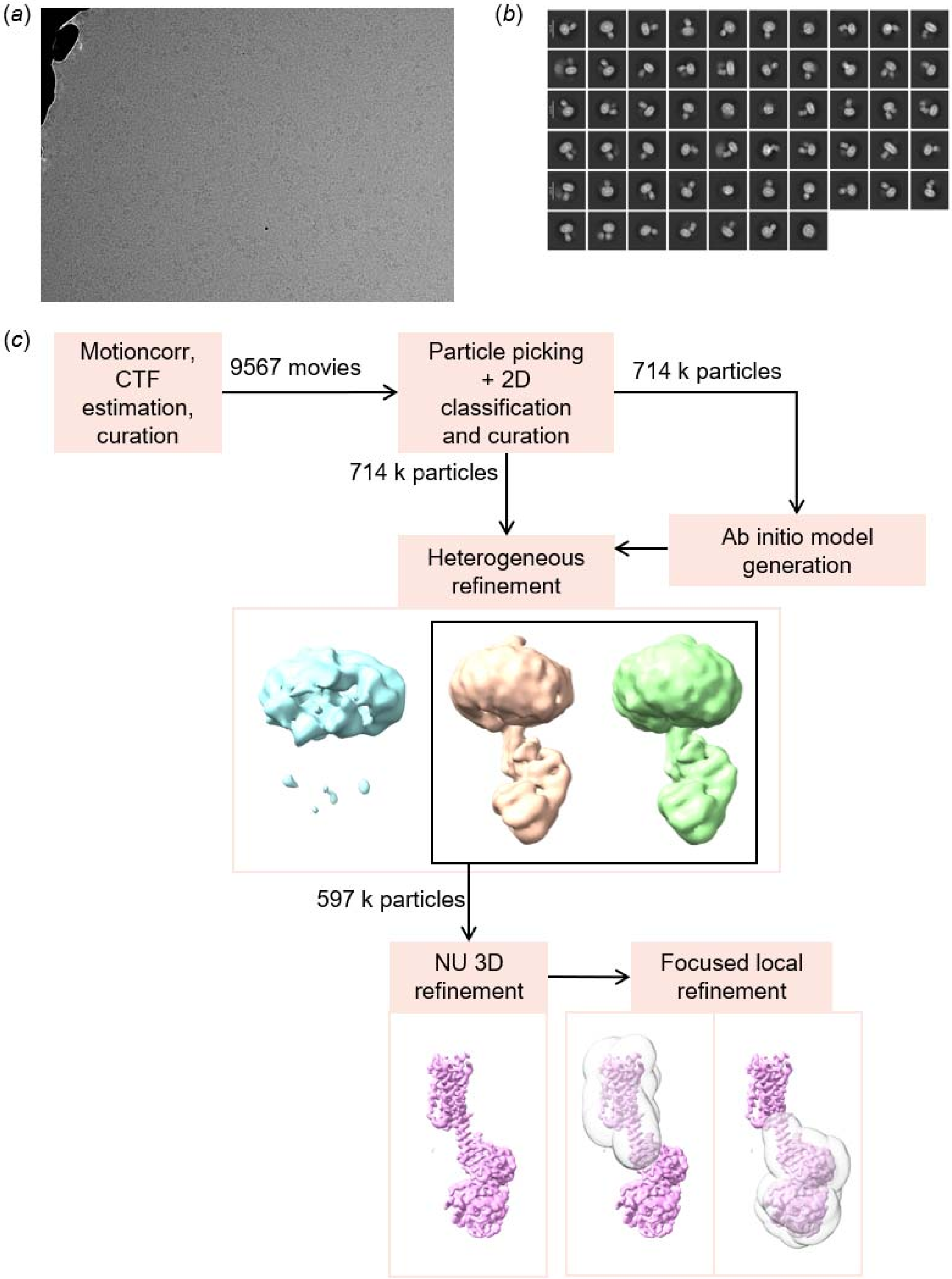

Figure S3 Cryo-EM data processing of A_{2A}R-beta-lactamase$_{Bli}$ + BLIPII + ZM241385 (*a*) Example micrograph, (*b*) Final selection of 2D class averages (representing 714k particles), (*c*) Data processing workflow: the output maps of the heterogeneous refinement step are shown in blue, orange, and green, with 2 classes selected for further refinement (orange, green); Focused local refinement with masks (grey) around the A_{2A}R region (left) or the beta-lactamase and BLIPII region (right).

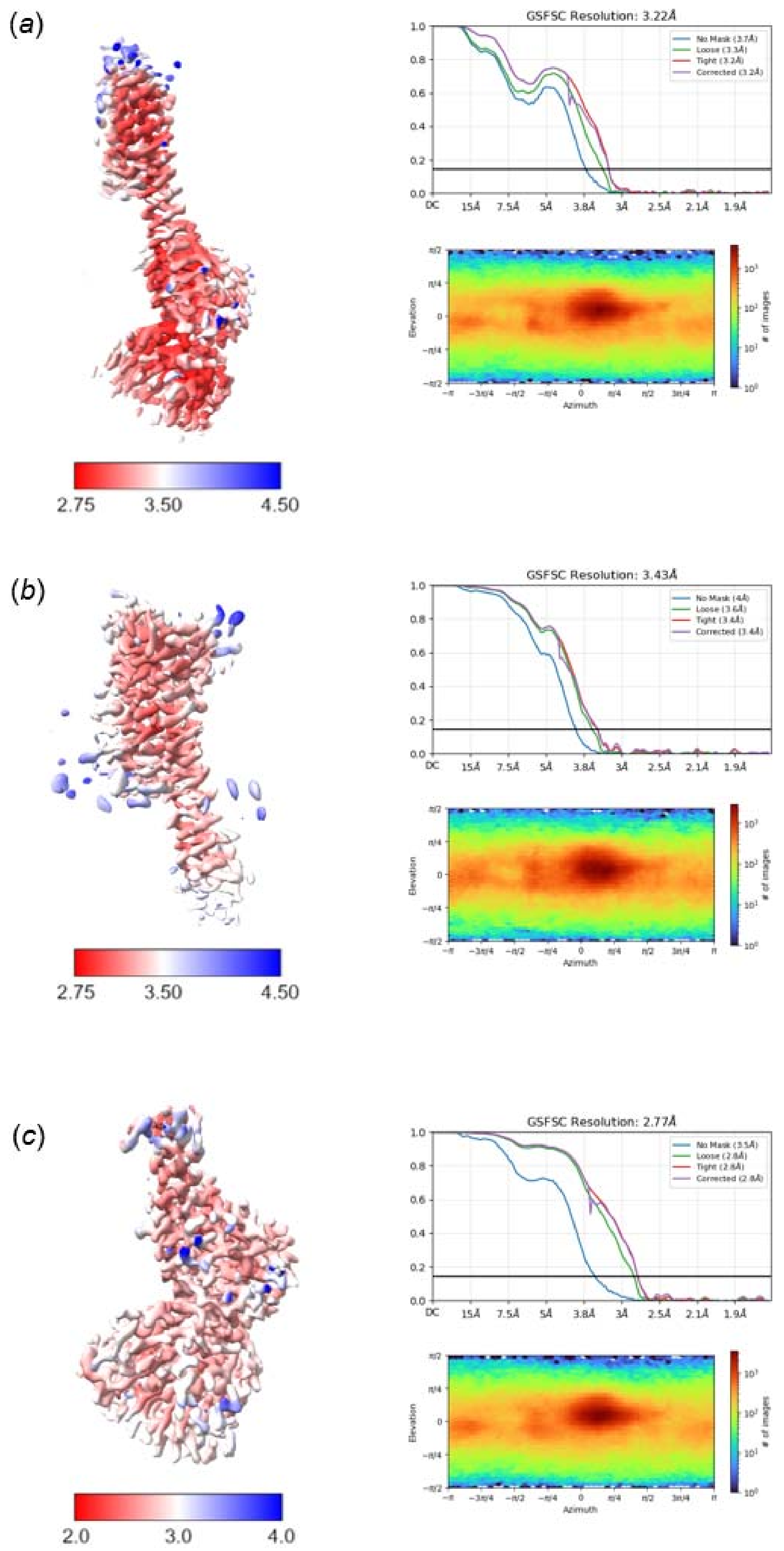

Figure S4 Local resolution-filtered density map, coloured by local resolution estimates, FSC curve, and angular orientations heat map of (*a*) overall consensus map before focused local refinement steps (map threshold at 0.476), (*b*) after A_{2A}R-focused local refinement step (map threshold at 0.476), and

(*c*) after beta-lactamase$_{Bli}$ + BLIPII-focused local refinement step (map threshold at 0.300). Estimated local resolution is indicated by the red to blue bar.

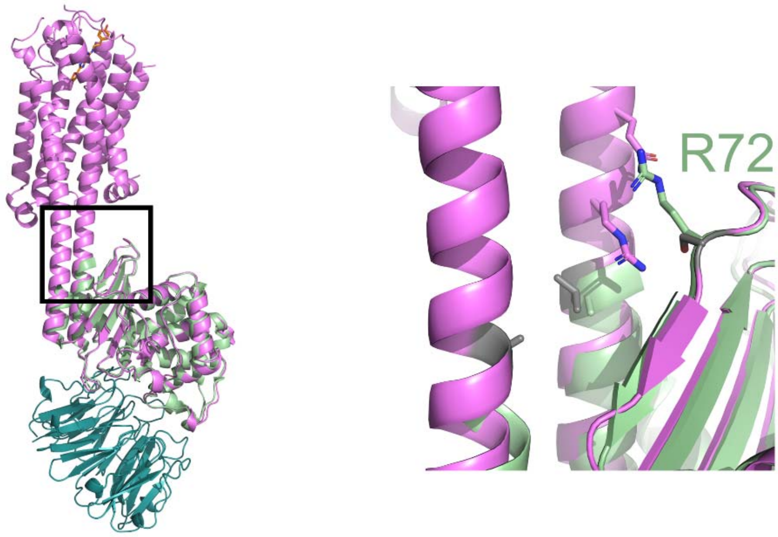

Figure S5 Comparison of A_{2A}R-betalactamase$_{Bli}$ fusion region of the cryo-EM structure (this study, pink and teal) with the beta-lactamase$_{Bli}$ crystal structure (PDB ID 1I2S (Fonze *et al.*, 2002), green model), showing mutations in grey (M46A, R72S, A301L) on the cryo-EM model, highlighting R72S of beta-lactamase$_{Bli}$.

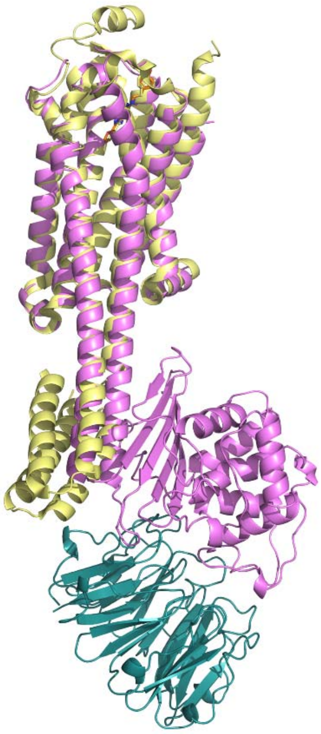

Figure S6 Overlay of the cryo-EM structure of ZM241385-bound A_{2A}R-betalactamase$_{Bli}$ + BLIPII (this study, pink and teal) and X-ray crystal structure of A_{2A}R with a bRIL inserted into ICL3 bound to ZM241385 (PDB 5IU4 (Segala *et al.*, 2016), yellow), aligned on A_{2A}R, showing a similar overall fold (r.m.s.d. = 1.00 Å over the A_{2A}R region of the cryo-EM structure).
